# Multisystem Inflammatory Syndrome in Adults-Related Unstable Angina With Coronary Aneurysm in a Young Female: A Case Report

**DOI:** 10.7759/cureus.56162

**Published:** 2024-03-14

**Authors:** Hiroki Aihara

**Affiliations:** 1 Department of Cardiovascular Medicine, Abashiri-Kosei General Hospital, Abashiri, JPN

**Keywords:** multisystem inflammatory syndrome in adults (mis-a), kawasaki-like multisystem inflammatory disease, coronary artery bypass graft surgery, covid-19, coronary artery aneurysms

## Abstract

Multisystem inflammatory syndrome in adults (MIS-A) is a systemic inflammatory disease associated with COVID-19 and follows coronary artery aneurysms similar to Kawasaki disease. In many cases, it is improved by treatments such as high-dose steroids or intravenous immunoglobulin (IVIg). However, the role of untreated coronary artery aneurysms leading to future stenosis remains unknown. Untreated MIS-A may potentially lead to the formation of coronary aneurysms. In cases of COVID-19 where young adults present with angina-like symptoms, an evaluation for angina is considered. Herein, we report a case of a 27-year-old female who developed unstable angina with coronary artery aneurysms six months after COVID-19 infection. She required surgery for unstable angina, which resulted in an improvement in chest pain. Coronary artery lesions are considered to be related to MIS-A, and treatment was conducted in accordance with that for Kawasaki disease. Currently, the pathological differences and prognosis between MIS-A and Kawasaki disease remain unclear, but the elucidation of the conditions is warranted in the future.

## Introduction

Multisystem inflammatory syndrome in adults (MIS-A) is a syndrome that presents with a Kawasaki disease-like reaction following COVID-19 infection [1] and can often lead to coronary artery dilatation or the formation of giant aneurysms. Many cases are reported during the acute phase when patients present with cardiogenic shock and are subsequently observed for coronary aneurysm regression following treatment with high-dose steroids or intravenous immunoglobulin (IVIg) therapy [2]. However, whether once-formed coronary aneurysms can lead to acute coronary syndromes similar to Kawasaki disease sequelae in the long term remains unknown. Herein, we report the case of a young female who developed typical angina approximately six months after COVID-19 infection and was diagnosed with unstable angina due to coronary aneurysms, ultimately requiring surgical treatment.

## Case presentation

A 27-year-old female with no known past medical history presented with chest pain radiating from the shoulder to the back and chest tightness during light exertion. She had been infected with COVID-19 six months before and had no history of Kawasaki disease or smoking with any family history of coronary artery disease or sudden death. She received three SARS-CoV-2 vaccine doses, with the last dose administered nine months prior to the infection. Her creatine kinase and brain natriuretic peptide levels, X-ray, electrocardiogram, and echocardiography revealed no abnormalities, but a troponin I level of 401.6 pg/mL (normal: <26.2 pg/mL) was observed, indicating mild myocardial injury. Thyroid hormones, C-reactive protein, erythrocyte sedimentation rate, and antinuclear, anticardiolipin, and antineutrophil cytoplasmic antibodies were negative.

**Figure 1:**
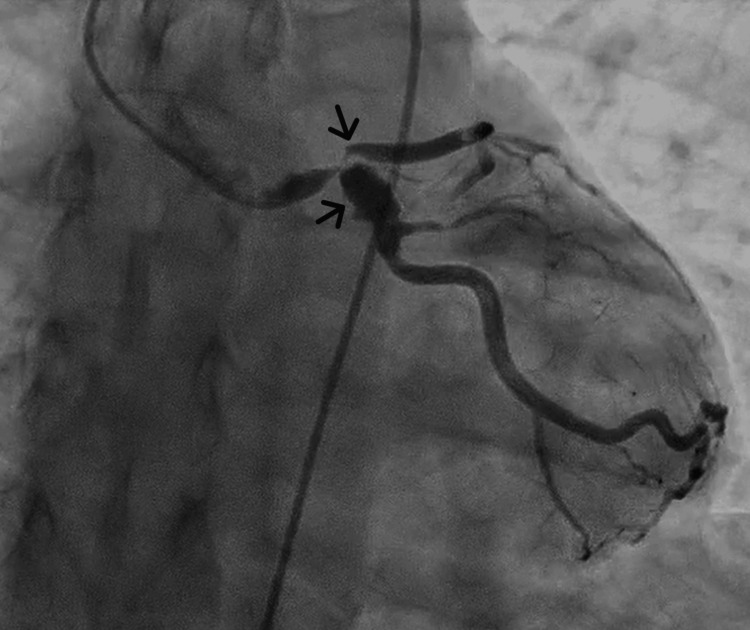
Results of the coronary angiography of the left coronary artery A 99% stenosis with delayed contrast in the proximal left anterior descending artery (LAD) and a 90% stenosis with a coronary aneurysm in the proximal left circumflex artery (LCX)

**Figure 2:**
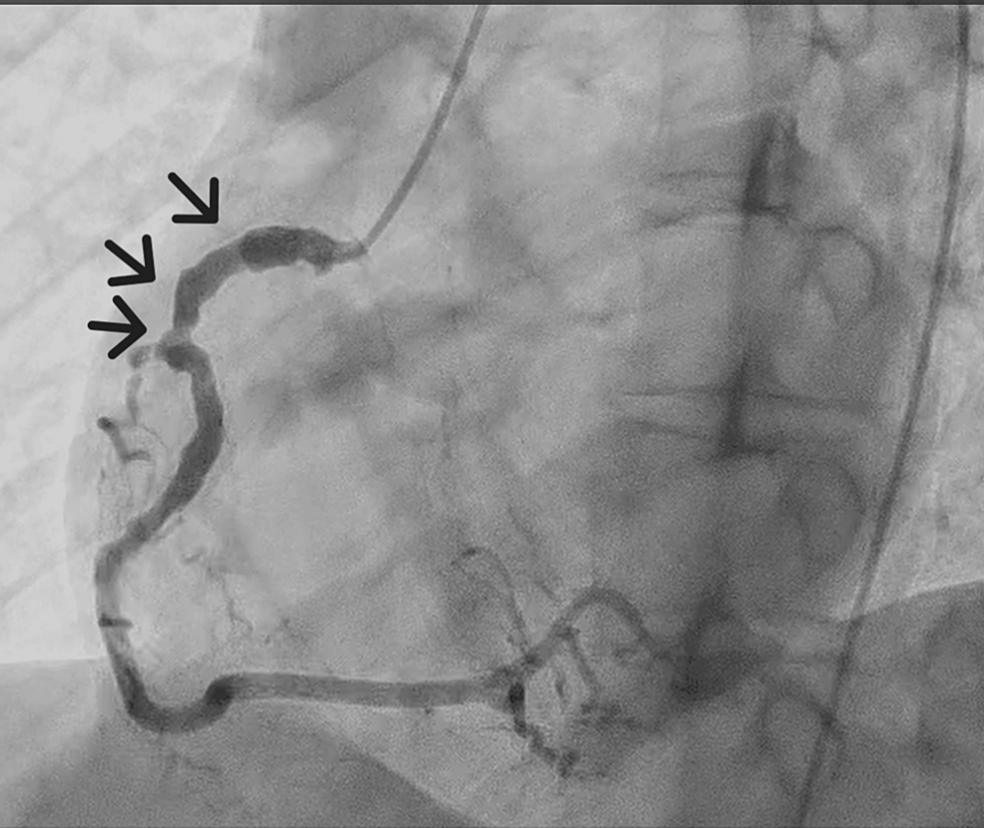
Results of the coronary angiography of the right coronary artery (RCA) A 50% stenosis with coronary artery aneurysm regression in the proximal RCA

Coronary angiography was performed after considering COVID-19-induced endothelial injury or severe coronary vasospasm. Coronary angiography revealed a 99% stenosis with delayed contrast in the proximal left anterior descending artery (LAD), and a 90% stenosis with a coronary aneurysm was observed in the proximal left circumflex artery (LCX) (Figure 1). A 50% stenosis was observed in the proximal right coronary artery (RCA) (Figure 2); it was dilated from the proximal to middle segment, with areas showing coronary artery aneurysm regression. Collateral circulation from the posterior descending artery of the RCA to the septal branches of the LAD was observed. She was diagnosed with unstable angina in two locations (the LAD and LCX).

**Figure 3:**
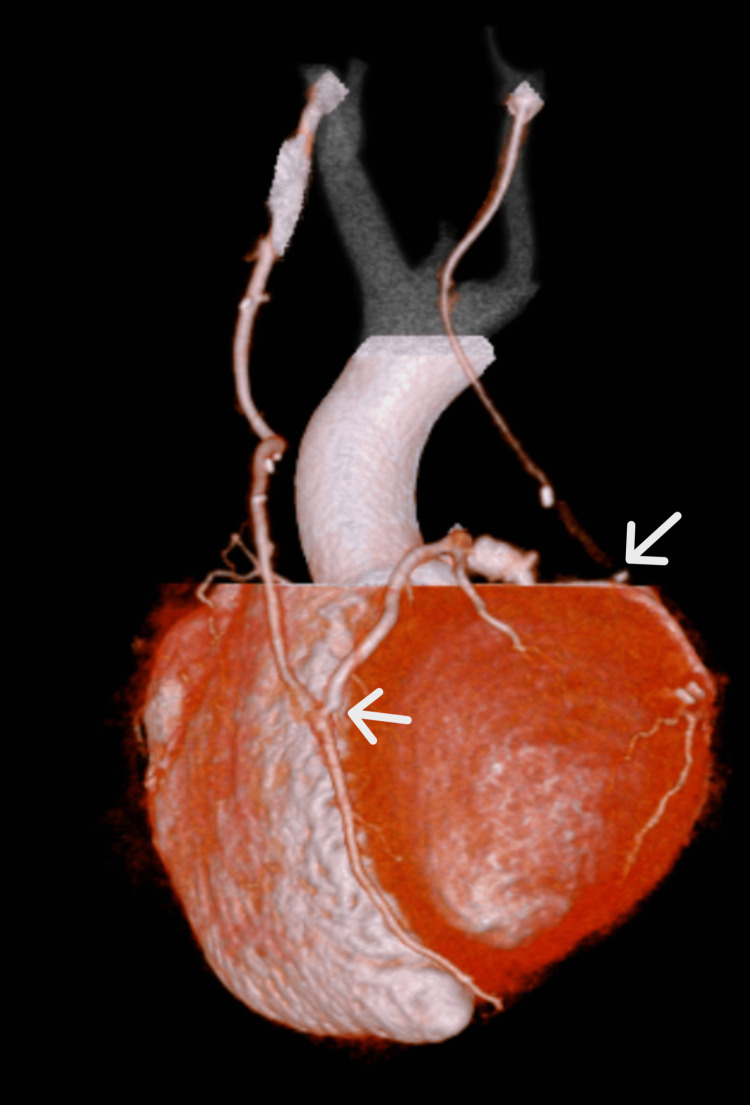
A reconstructed image from contrast-enhanced computed tomography after coronary artery bypass graft Bypass grafting has been performed from the right internal mammary artery to the LAD and from the left internal mammary artery to the LCX, with good patency of the vessels LAD, anterior descending artery; LCX, left circumflex artery

Beating-heart coronary artery bypass grafting was performed in two branches (the right internal mammary artery to the LAD and the left internal mammary artery to the posterior lateral branch of the LCX). In the subacute phase after coronary artery bypass, contrast-enhanced computed tomography was performed, and the patency of the bypass grafts was confirmed (Figure 3). Postoperatively, chest pain observed during light exertion improved, and the patient was able to walk short distances without dyspnea. The patient was discharged 22 days after surgery.

## Discussion

The persistent malaise following COVID-19 infection is commonly referred to as long COVID, which is known to cause symptoms in the cardiovascular and respiratory systems and throughout the body. Since the outbreak of COVID-19 in 2019, multisystem inflammatory syndrome in children (MIS-C), which causes systemic inflammatory symptoms similar to Kawasaki disease post COVID-19, has been reported in children [1]. Subsequently, similar symptoms have been reported in adults and have come to be recognized as MIS-A.

Coronary artery aneurysms may develop after COVID-19, often alongside MIS-A, involving severe systemic inflammation impacting multiple organs. Approximately 8%-9% of MIS-A cases report coronary artery aneurysms [2,3]. Early intervention with high-dose steroids or intravenous immunoglobulin improves many cases. The incidence of coronary artery aneurysms in patients not progressing to MIS-A remains unexplored. While systematic reviews and meta-analyses show cardiovascular complications persisting over four weeks post COVID-19, including 9.79% chest pain (95% CI: 7.24-13.11), 1.27% myocardial infarction, and 0.41% coronary artery disease [4], no study has examined the presence of coronary artery aneurysms. Here, we report a case of acute coronary syndrome with a coronary artery aneurysm post COVID-19, treated surgically following Kawasaki disease protocol.

Kawasaki disease is recognized for post-disease inflammation causing coronary arteritis, often subsiding around the 40th day, with most aneurysms decreasing in size during recovery. Coronary artery aneurysms elevate thrombosis risk due to blood flow disturbances and endothelial dysfunction, with most acute myocardial infarctions occurring within two years of Kawasaki disease onset [5,6]. Even when coronary aneurysms regress, the persistence of pathological organic changes and endothelial dysfunction has been established. As a result, there have been various reports leading to acute myocardial infarction. In the present case, the patient contracted COVID-19 for more than six months, and there was no history of MIS-A. While the pathological evidence linking this condition to COVID-19 is scarce, the absence of a Kawasaki disease history and the patient's age in the late 20s make a recent onset of Kawasaki disease unlikely. Therefore, the possibility of COVID-19-induced coronary aneurysms and associated unstable angina was suspected. Though the pathological variances between Kawasaki disease and MIS-A are yet to be fully understood, we hypothesize that regressing coronary artery aneurysms underwent abnormal remodeling, leading to severe stenosis.

## Conclusions

It is advisable to consider MIS-A as a part of the differential diagnosis while treating systemic malaise persisting after COVID-19 infection. In cases of chest pain following COVID-19 infection, it is crucial to evaluate for myocardial damage. If observed, coronary examination including contrast-enhanced computed tomography and coronary angiography should be considered, if necessary. MIS-A presents with a condition similar to the sequelae of Kawasaki disease, but the pathological differences and prognosis remain unknown. Further elucidation of the condition, including the sequelae of COVID-19, is warranted in the future.

## References

[1] Kabeerdoss J, Pilania RK, Karkhele R, Kumar TS, Danda D, Singh S (2021). Severe COVID-19, multisystem inflammatory syndrome in children, and Kawasaki disease: immunological mechanisms, clinical manifestations and management. Rheumatol Int.

[2] Feldstein LR, Rose EB, Horwitz SM (2020). Multisystem inflammatory syndrome in U.S. children and adolescents. N Engl J Med.

[3] Diakite S, Bousdira N, Tachon G, Ackermann F, Groh M, Rohmer J (2021). Regression of coronary aneurysms with intravenous immunoglobulins and steroids for COVID-19 adult multisystem inflammatory syndrome. JACC Case Rep.

[4] Guo B, Zhao C, He MZ (2023). Identifying patterns of reported findings on long-term cardiac complications of COVID-19: a systematic review and meta-analysis. BMC Med.

[5] Suzuki A, Miyagawa-Tomita S, Nakazawa M, Yutani C (2000). Remodeling of coronary artery lesions due to Kawasaki disease: comparison of arteriographic and immunohistochemical findings. Jpn Heart J.

[6] Fukazawa R, Kobayashi T, Mikami M (2017). Nationwide survey of patients with giant coronary aneurysm secondary to Kawasaki disease 1999-2010 in Japan. Circ J.

